# The Enigma of Recurrent Lung Abscess: Management and Outcomes in a School-Aged Child With a Review of Literature

**DOI:** 10.7759/cureus.63579

**Published:** 2024-07-01

**Authors:** Vishal V Bhende, Jigar P Thacker, Deepakkumar V Mehta, Mathangi Krishnakumar, Birva N Khara

**Affiliations:** 1 Pediatric Cardiac Surgery, Bhanubhai and Madhuben Patel Cardiac Centre, Shree Krishna Hospital, Bhaikaka University, Karamsad, IND; 2 Pediatrics, Pramukhswami Medical College, Shree Krishna Hospital, Bhaikaka University, Karamsad, IND; 3 Radiodiagnosis and Imaging, Pramukhswami Medical College, Shree Krishna Hospital, Bhaikaka University, Karamsad, IND; 4 Anesthesiology, St. John’s Medical College Hospital, Bengaluru, IND; 5 Anesthesiology, Pramukhswami Medical College, Shree Krishna Hospital, Bhaikaka University, Karamsad, IND

**Keywords:** antibiotics, lung abscess, percutaneous ultrasonography guided trans-thoracic needle aspiration, thoracoscopy, thoracotomy, video-assisted thoracoscopic surgery (vats)

## Abstract

Pediatric lung abscess is a rare and poorly studied disease entity. In the past, prolonged courses of intravenous (IV) antibiotics have been successfully used; however, with the advent of interventional radiology, the main therapeutic approach is through percutaneous placement of pigtail catheters with ultrasound and computed tomography (CT) direction, where available. The pathogen yield identified from fluid samples of the abscess has dramatically increased owing to the greater invasive measures, such as aspiration and drainage, as well as enhanced microbiological diagnostic methods, which also include polymerase chain reaction testing.

In our case report, in 2012 when the patient was two years old, she was diagnosed with pulmonary Koch's and underwent anti-Koch's therapy, category 2. High-resolution CT of the chest revealed a large lobulated cavitary lesion with an air-fluid level suggestive of a right lung abscess. After initial therapy with IV antibiotics for three weeks and a negative tuberculosis work-up, she underwent right limited lateral thoracotomy and drainage with decortication of the right lung abscess (LA) in 2019 via a left endobronchial tube with a bronchial blocker (general endobronchial anesthesia). All samples sent for histopathologic examination after surgery yielded negative results, and she was discharged after a course of injectable antibiotics for 21 days. She remained almost symptom-free for the next four years. Thereafter, she presented with a right LA recurrence due to a thick-walled cavitary lesion, with a severely damaged right lower lung lobe resulting in right lower lobectomy under single-lung ventilation (double-lumen endotracheal tube No. 26 Fr.). Culture results should guide management, particularly for immunocompromised patients, as the LA may be attributed to complications arising from underlying conditions. Primary lung abscesses (PLA) in children are typically caused by *Staphylococcus aureus, Streptococcal species,* and *Klebsiella pneumoniae*. Compared to adults, children with PLA and secondary lung abscesses have a meaningfully greater rate of recovery.

## Introduction

The suppuration and necrosis of the lung parenchyma (LP) results in the creation of a narrow cavity that has reinforced walls containing purulent substance, referred to as an LA [1,2]. In children, LA can be classified as primary lung abscess (PLA) (no predisposing factors involved) or secondary (systemic or pulmonary predisposing factors involved). Lung abscesses can also be classified as multiloculated, uniloculated, aspirational, hematogenous, putrid, and non-putrid (aerobic and anaerobic) [3]. Other classifications depend on the illness duration, that is, acute, which lasts four or fewer weeks, or, even chronic which lasts for more than four weeks [4]. PLA is reported to be caused commonly by *Streptococcus pneumoniae* and *Staphylococcus aureus*, whereas anaerobes (27%), *Pseudomonas aeruginosa* (13%), *S. aureus* (13%), *S. pneumonia* (7%), *Hemophilus influenza* (7%), *Escherichia coli*, or *Klebsiella* are the common causes of secondary LA. Lung diseases that affect the anatomy and/or physiology, such as congenital lung malformations, ciliary disorders, cystic fibrosis, immunodeficiencies, aspirations, or infections, can also result in secondary lung abscesses. Although uncommon, tuberculosis can further complicate lung abscesses [5]. The pathophysiology is frequently initiated by lung inflammation, which then progresses to cell death, leading to fibrosis, and cavitations, causing suppurative invasion and damage of the LP with central cavitation. Patradoon-Ho and Fitzgerald reported that the incidence of pediatric LA could be as low as 0.7/100,000 admissions/annually [6]. Similarly, Madhani et al. reported an incidence of 39 cases/10 years, rendering it a relatively uncommon pediatric illness [3]. The morbidity associated with pediatric LA is negligible compared to that of adults [7]. Although it is unusual among neonates, it is commonly encountered during the fall and winter seasons [8]. Chest X-rays, ultrasonography, and computed tomography (CT) can establish the diagnosis.

Respiratory culture usually provides a definitive diagnosis, although not in all cases, and the first line of treatment is prolonged empiric broad-spectrum antibiotic treatment. Fortunately, interventional radiology offers another treatment option where a catheter can be safely and effectively placed in the lung. Invasive methods comprise drainage, aspiration, and resection [6]. In this case scenario, the emphasis is on the LA evolution leading to recurrence and offering lobectomy as the treatment modality with good outcomes in a school-aged child. Though invasive in nature this has the potential to offer completion of treatment thereby reducing disease burden and risk of recurrence which is a possibility with other lines of management.

## Case presentation

This case study was permitted by the Institutional Ethics Committee (IEC), H. M. Patel Centre for Medical Care and Education, Gujarat (approval no. IEC/BU/2023/Cr.21/135/2023, dated June 5, 2023) and the informed consent was signed and collected from the patient's parents prior to lung resection, which allowed us to use patient's data. The procedure concerning human participants fulfilled the ethical standards of the IEC and the 1964 Declaration of Helsinki and its succeeding modifications or similar ethical standards.

A 12-year-old school-aged girl was brought for consultation at the pediatric chest clinic of our hospital with complaints of recent-onset, productive, moderate-degree cough associated with seasonal variation and a history of moderate-grade fever. Her vital signs were as follows: temperature of 100.8°F (febrile), heart rate of 120 beats/min, blood pressure of 110/72 mm Hg, and respiratory rate of 22 breaths/min. There was no history of chest pain, sense of vomiting, hemoptysis, vertigo, sudden or sustained loss of weight, chills, excessive sweating at night, recent travel, or history of contact with any sick person. On examination, decreased breath sounds were noted in the right lower zone with bilateral crackles, which were accentuated in the right upper zone.

Her laboratory investigations through her course of admission are depicted in Table 1. She presented with elevated C-reactive protein (CRP) which settled with treatment, her other investigations were unremarkable. The blood culture did not isolate any organism after five days of aerobic incubation.

**Table 1:** Summary of the patient's laboratory data on admission, surgical intervention till discharge POD: post-operative day; WBC: white blood cell

Test	Patient results					Reference range
	Day of admission 0	Day 10	Day 20	Day after surgery (POD 1)	Day after surgery (POD 7)	
Hemoglobin	11.1	9.7	9.5	8.2	10.2	11.1-14.1 g/dl
Total leucocyte count (WBC count)	11.7	10.7	26.9	21	13.8	6-16 × 1000/ul
C-reactive protein (CRP)	254.5	89.5	-	-	76.6	<3 mg/l
Hematocrit	34.7	29.5	25.7	22.2	32.9	36-46%
Platelet count	395	852	287	242	478	150-450 × 1000/ul

The patient had a significant past history, diagnosed with pulmonary Koch's in 2012 at the age of two years and medicated with anti-Koch's therapy, category 2, although the patient was labeled as a drug defaulter. High-resolution computed tomography (HRCT) of the chest revealed a large lobulated cavitary lesion with an air-fluid level suggestive of a right lung abscess.

**Figure 1:**
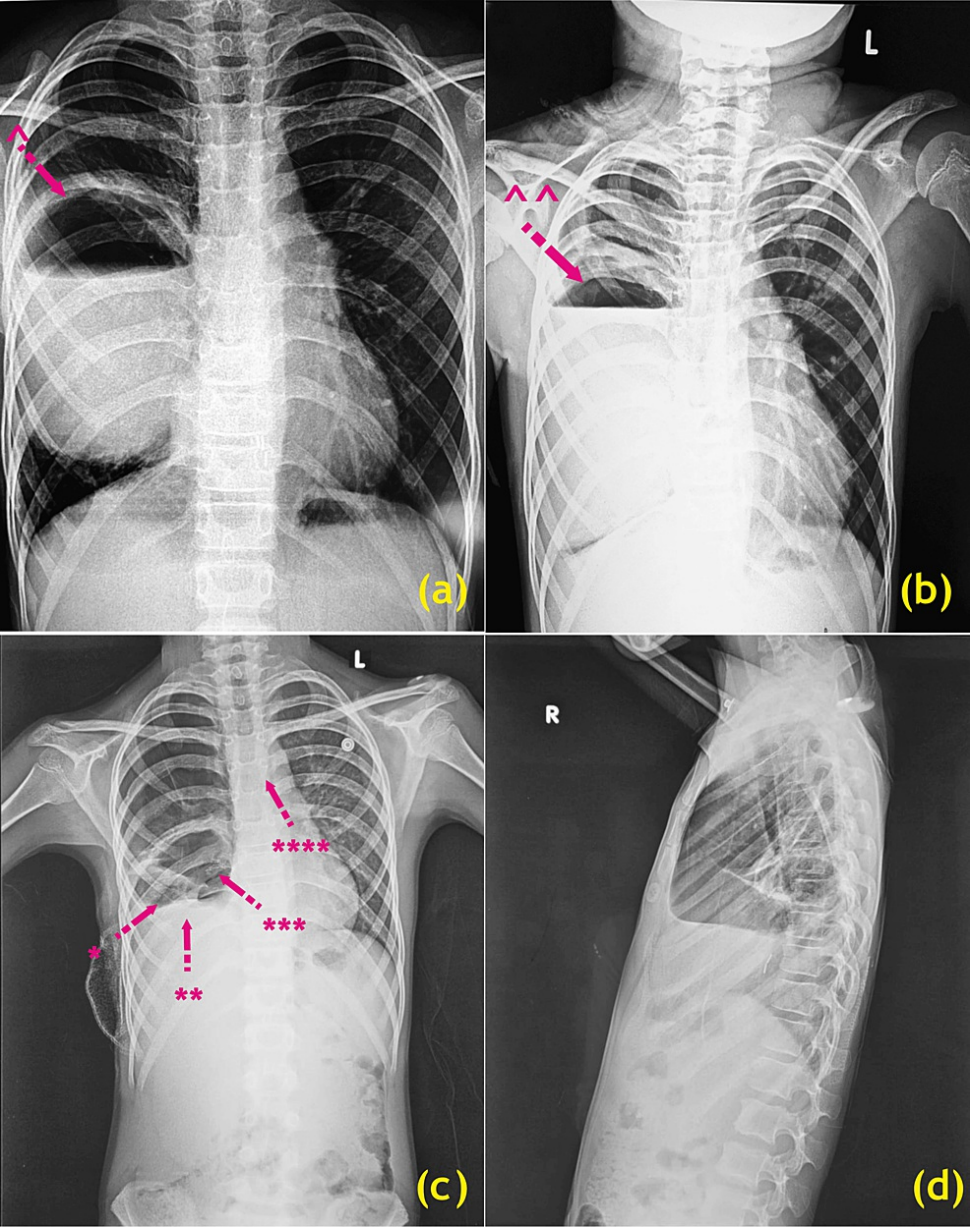
Evolution of recurrent right lung abscess. Chest X-ray pre-operative post-surgical status decortication of the right lung abscess under limited right lateral thoracotomy (a) initial stage, (b) advanced stage; chest X-ray post-operative, (c) chest X-ray standing PA view, and (d) chest X-ray right lateral view after right lower lobectomy of the lung at the time of discharge ^: right lung abscess with air-fluid level; ^^: right lung abscess with air-fluid level; *: right hemidiaphragm (S/P Bruver’s maneuver); **: original position of right hemidiaphragm; ***: right lower lobe bronchial stapler; ****: emerging scoliosis S/P: status post Image Credits: Dr. Vishal V. Bhende

**Figure 2:**
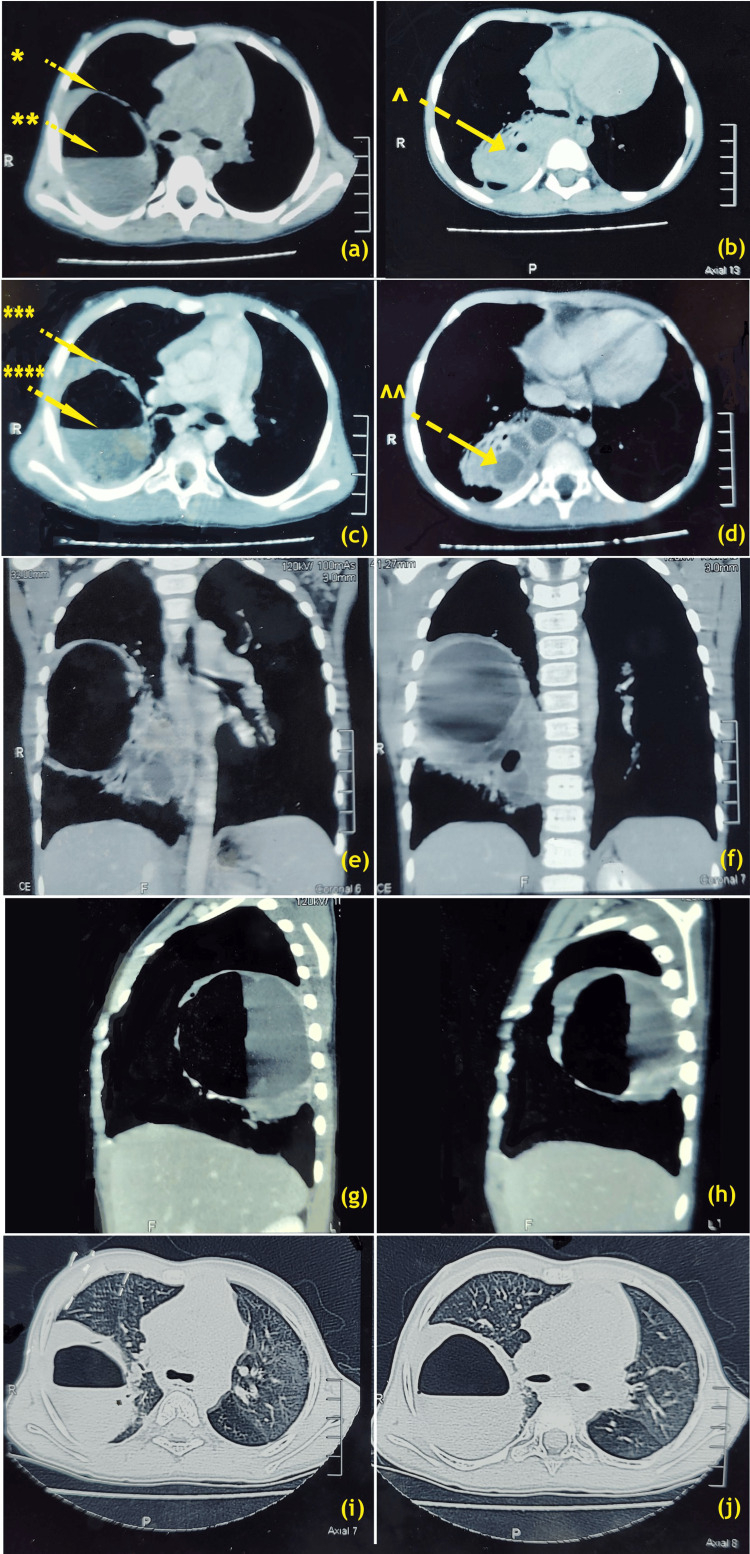
Evolution of recurrent right lung abscess. Pre-decortication surgery stage, plain CT scan axial images (a) *: thick-walled big lung cavity; **: air-fluid level in big cavity, (b) ^: small lung cavities with fluid contrast CT scan axial images, (c) ***: enhancing thick-walled big lung abscess; ****: air-fluid level in abscess, (d) ^^: small lung abscesses with enhancing walls, (e & f) contrast CT scan coronal reconstructions, (g & h) contrast CT scan right para-sagittal reconstructions, (i & j) plain CT scan axial images at carinal levels thick walled big lung cavity with air-fluid level in lung window setting Image Credits: Dr. Deepakkumar V. Mehta

**Figure 3:**
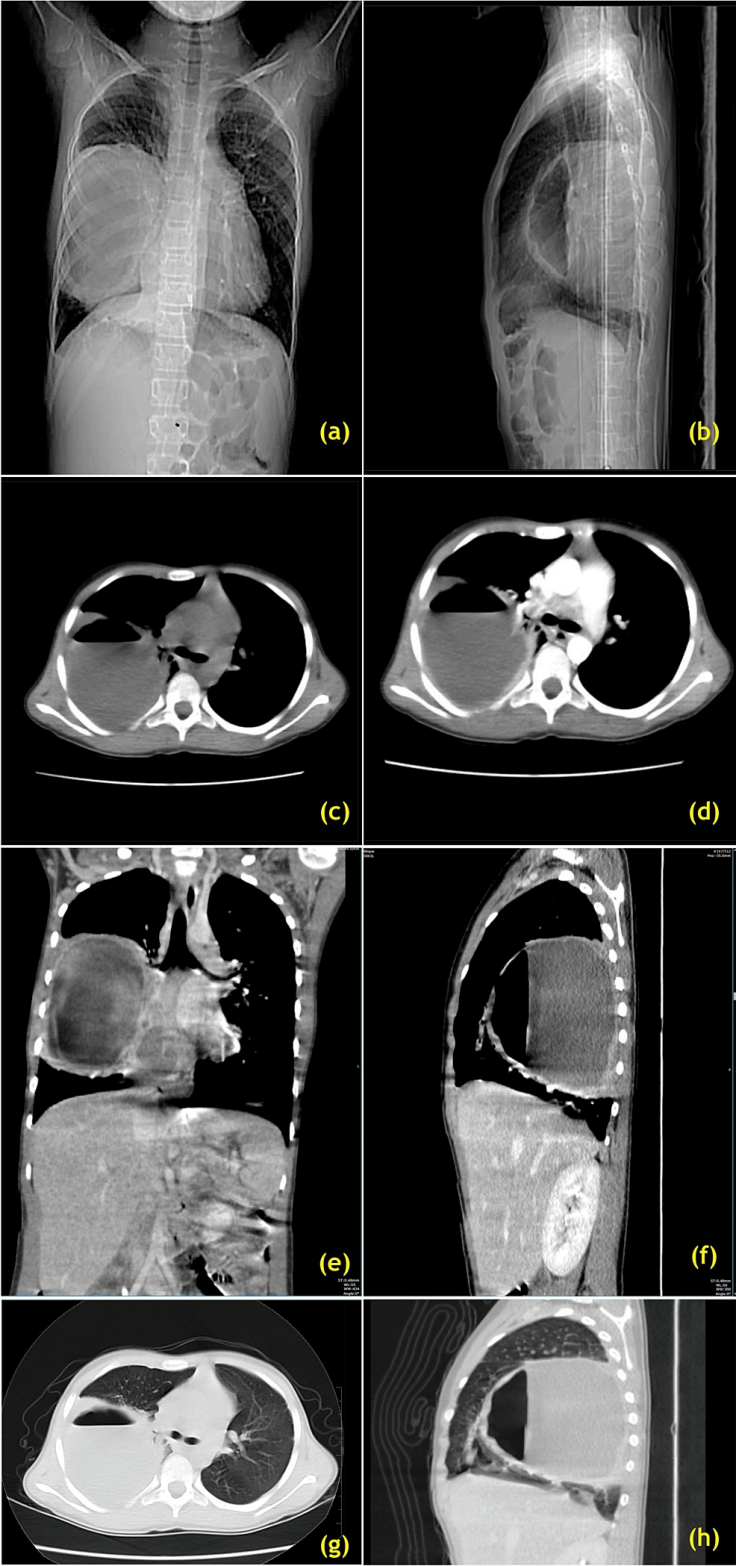
Evolution of recurrent right lung abscess. Pre-right lower lobectomy surgery stage (a) CT scanogram AP view, (b) CT scanogram right lateral view, (C) plain CT axial image at carinal level, (d) contrast CT axial image at carinal level, (e) contrast CT coronal reconstruction at carinal level, (f) contrast CT right para-sagittal reconstruction, (g) plain CT axial at carinal level lung window setting 5 mm, (h) lung window sagittal image AP: antero-posterior Image Credits: Dr. Deepakkumar V. Mehta

**Figure 4:**
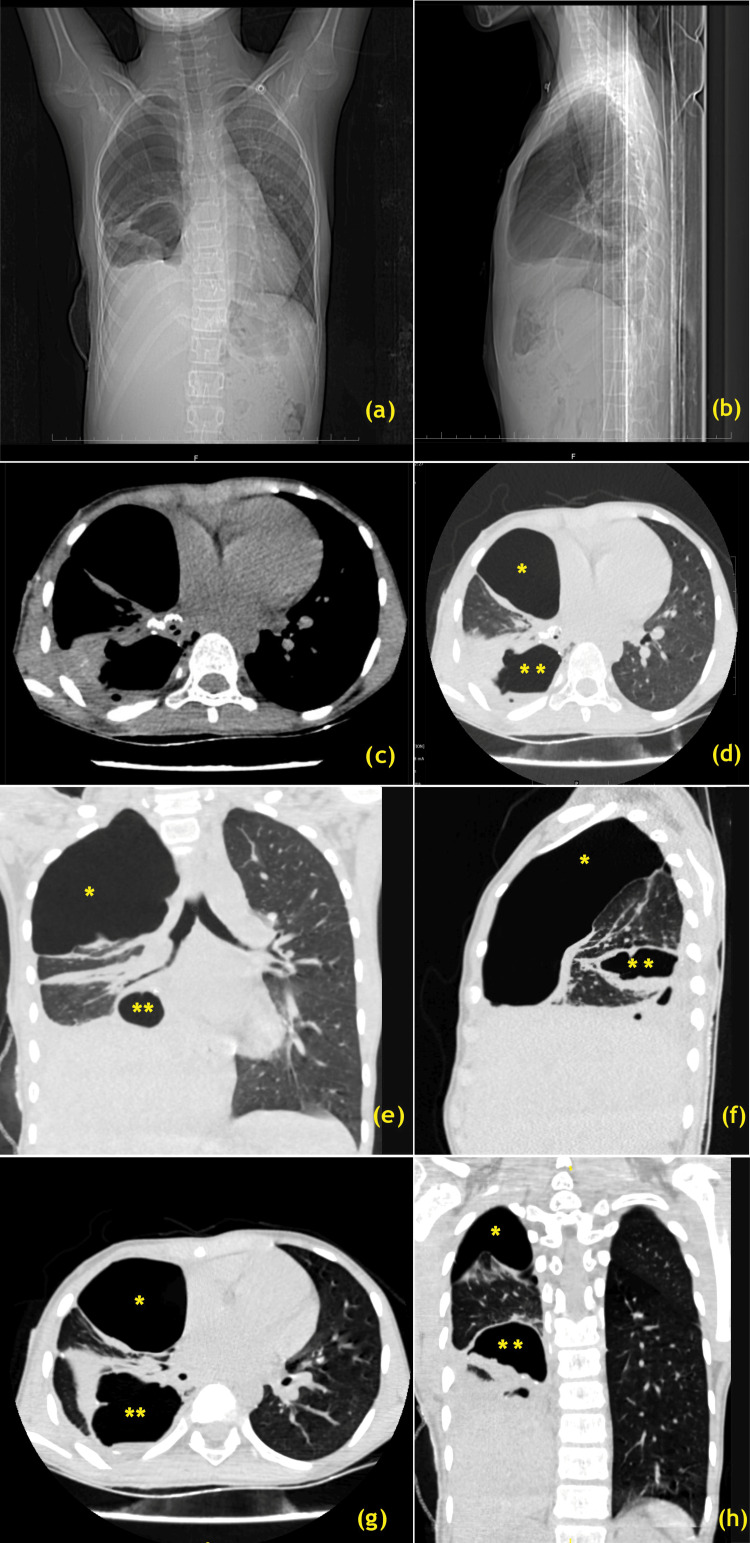
Evolution of recurrent right lung abscess. Post-right lower lobectomy surgery stage on discharge (a) CT scanogram AP view, (b) CT scanogram right lateral view, (c) axial images of lung bases at ventricular level mediastinal window setting, (d) HRCT lungs axial ventricular level lung window setting, (e) HRCT lungs coronal reconstruction at carinal level and HRCT lungs three plane reconstructions (f) right para-sagittal, (g) axial, and (h) posterior coronal lung window setting *: big pneumothorax; **: encysted hydro-pneumothorax AP: antero-posterior; HRCT: high-resolution computed tomography Image Credits: Dr. Deepakkumar V. Mehta

After a three-week initial therapy with intravenous (IV) antibiotics and a negative tubercular work-up, in 2019, she underwent right limited lateral thoracotomy and drainage with decortication of the right LA under a left endobronchial tube No. 5 Fr. with a right bronchial blocker (general endobronchial anesthesia) with the patient placed in left lateral position. Chest entered in the right 5th intercostal space. Dense adhesions of the right lung to the parietal pleura were noted which was gradually released. A thick peel noted over the lower part of the lung was peeled off with fibrinous flakes and purulent fluid present in the right costo-phrenic angle evacuated. All samples sent for histopathologic examination after surgery yielded negative results, and she was discharged after a course of injectable antibiotics for 21 days. She remained almost symptom-free for the next four years before having a right LA recurrence. The recurrent right LA diagnosis was established using a chest X-ray (Figure 1) done during this admission, which also showed worsening scoliosis. HRCT chest imaging is shown in Figures 2, 3, 4.

HRCT revealed a large, fairly defined, thick-walled (4.4 mm maximum wall thickness), multi-loculated, cavitary lesion with an air-fluid level and a few thin internal septations noted within the right lower lobe pushing the horizontal fissure upward and medially with a surrounding area of mild ground glass and fibrous septations. The thick-walled cavitary lesion has a maximum dimension of 9 cm (antero-posterior) × 7.3 cm (transverse) × 11.2 cm (supero-inferior).

**Figure 5:**
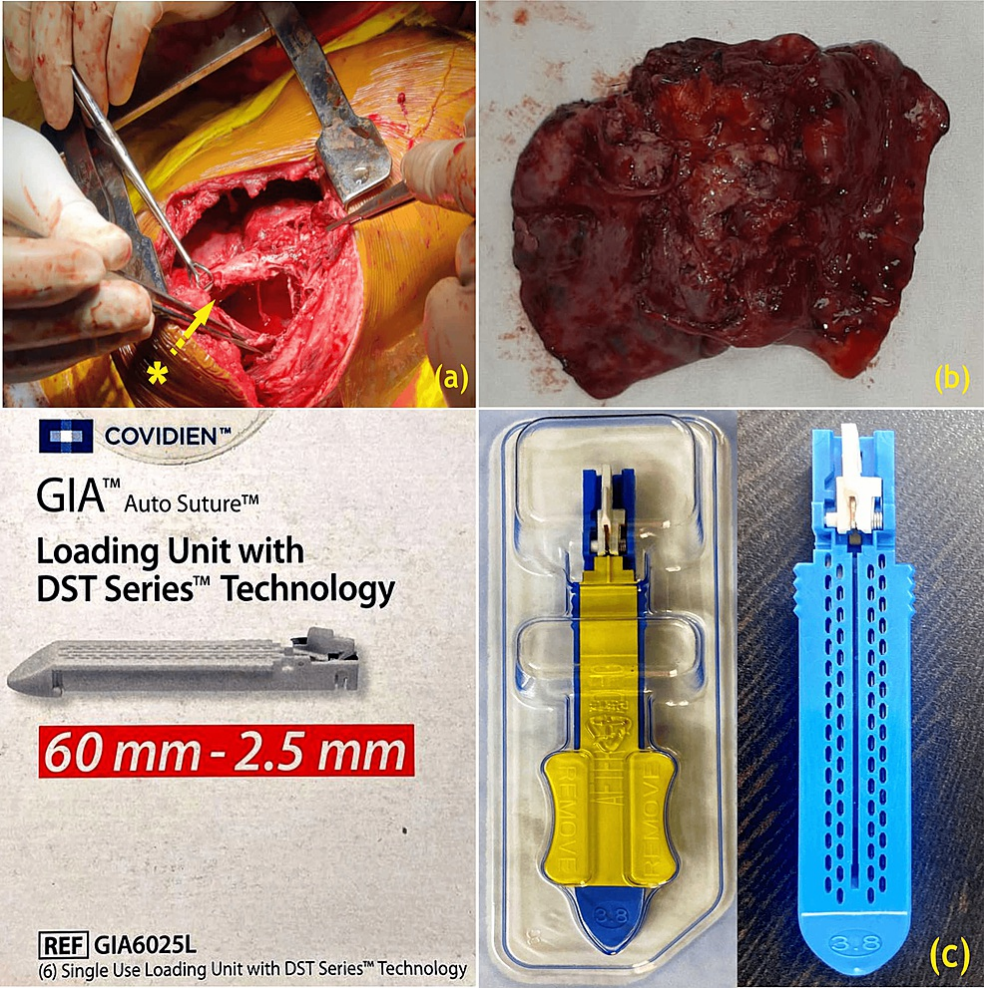
(a) Right postero-lateral thoracotomy chest entered through the 5th intercostal space (ICS), (b) specimen of the right lower lobe of lung delivered at surgery, (c) Covidien bronchial stapler (a) *: thick-walled cavitary lesion in the superior segment of the right lower lobe (right lung abscess); (c): permitted and attributed to Covidien GIA auto suture (https://images.app.goo.gl/aNybsV6Mpxof4kgj9) and Covidien GIA 10038L-GIA Auto Suture Loading unit DTS Series 100 mm (https://images.app.goo.gl/esqrTLzSAqn9Hj9H9) are under CC BY-NC-SA 4.0 (https://creativecommons.org/licenses/by-nc-sa/4.0/) Image Credits: Dr. Vishal V. Bhende

After optimization of the laboratory investigations, including the negative severe acute respiratory syndrome coronavirus 2 results, the patient was prepared for right lower lung lobectomy, with a right postero-lateral thoracotomy approach under single-lung ventilation (double-lumen endotracheal tube). Analgesia was administered via a thoracic epidural catheter through an epidural infusion of bupivacaine (0.25%) and fentanyl (1 mcg/cc), with a total dose of 10 ml (Figure 5).

**Figure 6:**
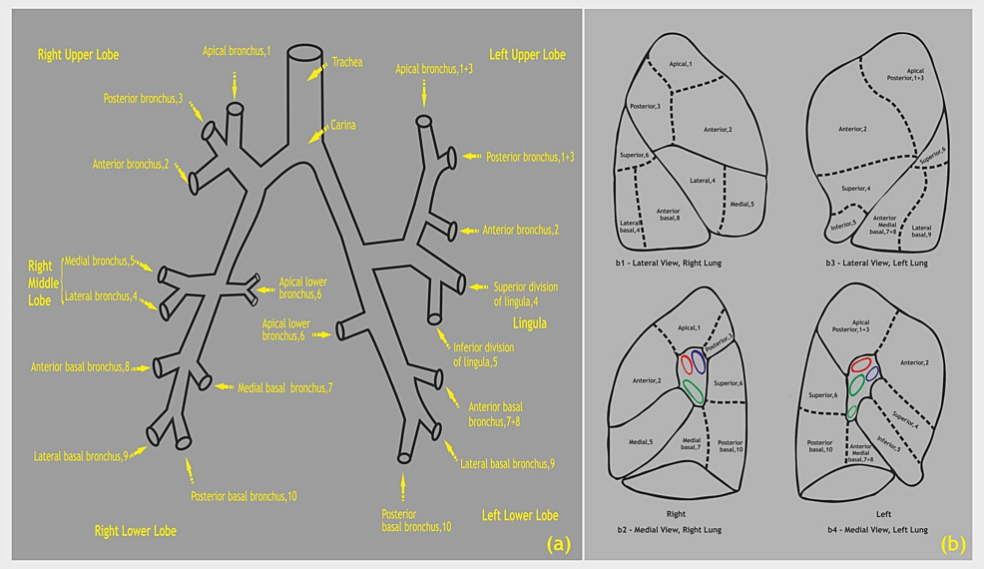
(a) Bronchopulmonary segmental nomenclature and numerical designations, (b) topographic positions of the bronchopulmonary segments of the right and left lung seen in lateral and medial views Image Credits: Dr. Vishal V. Bhende

The chest cavity was accessed through the right 5th intercostal space (ICS) as there was crowding of the ribs. The surgical outcome of an optimal lung resection depends on sound knowledge of the broncho-pulmonary segmental nomenclature (Figure 6).

**Figure 7:**
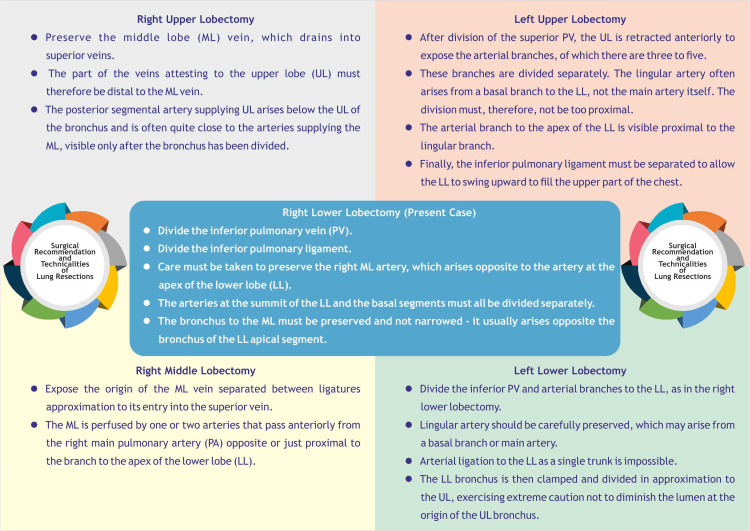
Surgical recommendation and technicalities of lung resections Image Credits: Dr. Vishal V. Bhende

The operative steps in our case (right lower lobectomy) and in general for other lung resections are summarized in Figure 7.

**Figure 8:**
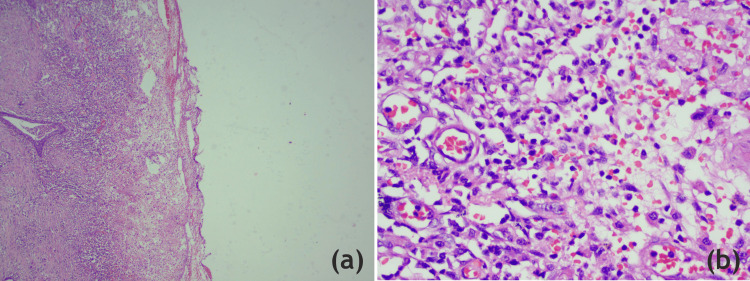
(a) 4x: the microphotograph shows the cavity wall with necrotic debris, intense suppurative inflammation, and changes in tissue repair underneath (H & E stain, 40x); (b) 40x: the microphotograph shows the cavity wall at higher magnification, emphasizing the polymorphonuclear infiltrate comprised predominantly of neutrophils with numerous thin-walled congested capillaries (a result of the angiogenesis going on) (H & E stain, 400x) H & E: hematoxylin and eosin Image Credits: Dr. Mustafa Ranapurwala

The right LL lung specimen was sent for histopathological examination (Figure 8).

The patient had an uneventful recovery with no operative or peri-operative complications. Blood loss was minimal and did not require transfusions after the redo-surgery. The patient was extubated on the table and discharged after a 21-day hospital stay. A major air leak was noted which subsided after 48-72 hours post-surgery. There was no chest asymmetry but a milder form of scoliosis was noted.

## Discussion

Limited literature exists elucidating pediatric lung abscesses [3]. Fernando et al. documented unjustified variations in managing pediatric LA with significantly longer hospital stays [9]. Some studies have found that the most predominant symptoms of LA are fever, cough, and dyspnea [10,11]. In the current case, the most prevalent symptoms were increased body temperature and cough, similar to previous reports [3,10,11]. Our patient had an initially dry, non-productive cough, which became productive when airway transaction was established [10,11].

Lung abscesses can occur anywhere in the lung [10]; in this case report, it was a right-sided lung abscess. There were no organisms identified, resulting in a negative blood culture report. The pathogen yield of lung abscesses might increase from approximately 30%-60% through the interventional approach [9,12,13].

More often, chest radiography is the preferred diagnostic approach, as in our case [3]. Chest CT, conversely, is usually recommended if the response is suboptimal prior to the intervention to differentiate LA from empyema and rule out latent lung malformations [11,13]. There were no bronchoscopy and lavage facilities at our institution.

The typical treatment protocol involves a prolonged duration of IV antibiotics for 24 days [4]. Interestingly, Chan et al. reported a mean IV antibiotic administration duration of 40 days [11]. Madhani et al. documented an IV antibiotic course of 28 days for PLA and 45 days for secondary LA [3]. Nevertheless, response to treatment, radiological progression, and acute phase reactants can be used to individualize combination therapies, as in our case [10,14].

IV antibiotic treatment can be unsuccessful if the LA is greater than 6 cm or if the patient has any co-morbidities or negative determinants [15]. Surgery may be another treatment alternative, that includes chest tube insertion, pigtail catheter placement, video-assisted thoracoscopy, and lobectomy [4,13].

We observed that our patient developed worsening scoliosis, as indicated by imaging results, following thoracic surgery. Post-surgery spinal deformities can be classified as “musculoskeletal deformity” in the Common Terminology Criteria for Adverse Events version 4.0 and as “other (specify)” in the Japan Clinical Oncology Group post-surgical complication criteria [16]. This study classified post-surgical thoracic and spinal deformities into pectus excavatum, pectus carinatum, scoliosis, and others. A deviation in Cobb angle of ≥10° on X-ray was designated as early scoliosis [17]. Previous studies have documented chest wall deformity developing post-surgery. In our patient chest wall abnormalities were found in the physical examinations and the imaging studies. It is important to look for other sequelae to thoracic surgery especially in children as this can significantly affect their growth, development, and quality of life. This approach ensures a precise assessment and systematic recording of the patient’s chest wall abnormalities, highlighting the importance of thorough documentation of findings from imaging.

Over time, the understanding of pathophysiology, treatment options and outcomes has improved. Table 2 summarizes the outcomes reported in previous studies ranging from the most common organisms isolated to the evolution of surgical management.

**Table 2:** Summary of pediatric lung abscess studies JCOG PC: Japan Clinical Oncology Group post-operative complications

Author and Year	Key Findings
Wali et al., 2002 [12]	Lung abscesses treated with antibiotics in 80-90% of cases. Pulmonary resection advised if conservative treatment fails.
Yen et al., 2004 [10]	23 children: 11 primary, 12 secondary lung abscesses. Pathogens isolated in 16 patients (69.6%). Most common: *Streptococcus pneumoniae*. Blood cultures positive in three patients (13.0%).
Chan et al., 2005 [11]	Early percutaneous aspiration crucial for pathogen identification. Primary abscesses: oral anaerobes, *Streptococcus aureus*. Secondary abscesses: consider gram-negative pathogens.
Yu et al., 2011 [15]	Managed with prolonged antibiotics (1-3 months). Poor prognostic factors: large abscess (> 6 cm), compromised immunity, neoplasm, advanced age, reduced consciousness, specific pathogens (*Klebsiella pneumoniae*, *Pseudomonas aeruginosa*, *Staphylococcus aureus*).
Katayama et al., 2015 [16]	JCOG PC criteria: 72 surgical adverse events (AEs) including gastroenterological, infectious, thoracic complications. Use 'other (specify)' if no applicable AE terms, with grading per Clavien-Dindo classification.
Kuhajda et al., 2015 [4]	Antibiotic therapy based on clinical and radiographic responses. Intravenous antibiotics: 5-21 days, followed by oral antibiotics for a total of 28-48 days, with periodic radiographic and laboratory monitoring.
Oliveira et al., 2015 [14]	Lung abscesses in pediatric patients are uncommon and often diagnosed late due to their insidious evolution. Chest radiography was the primary diagnostic test, while chest CT scans were useful for identifying predisposing or associated lung lesions and for guiding aspiration. The most frequent location for lung abscesses was the right lung, likely due to the anatomical position of the right main bronchus.
Madhani et al., 2016 [3]	Pediatric lung abscesses often polymicrobial: *Staphylococcus*, *Streptococcus spp.*, oral anaerobes. Surgery rarely needed unless prolonged fever or large abscess size.
Yang et al., 2016 [17]	Early-onset scoliosis (EOS) can impair pulmonary function due to spinal deformity and thoracic constraints during lung development.
Saito et al., 2020 [8]	Lung abscesses rare in infants, mostly treated with antibiotics. Surgery needed if deterioration despite one week of antibiotics.
Lee et al., 2022 [13]	Percutaneous catheter drainage (PCD) under imaging guidance effective for lung abscess treatment with low complication rate. Traversal of normal lung parenchyma risk factor for complications. Malignancy-related abscesses, major complications predict treatment failure.
Fernando et al.,2022 [9]	Surgery reserved for complications. Study aimed to describe pediatric lung abscesses and determine associations between presentation factors and outcomes.

Given the complexities and potential variations in the management of pediatric lung abscesses, our case underscores the need for standardized treatment protocols and comprehensive diagnostic approaches. The variability in management strategies suggests that a consensus on the best practices for treating pediatric LA is still lacking [9]. Our findings support the use of open resection as a valuable treatment option, particularly in cases where conservative management with prolonged IV antibiotics fails or is not feasible due to lesion size or patient co-morbidities. The success of our approach, combined with the literature supporting various surgical interventions, indicates that individualized treatment plans, guided by patient response and specific clinical circumstances, can lead to optimal outcomes. Future research should focus on establishing clear guidelines and exploring the long-term efficacy of different surgical techniques in managing pediatric lung abscesses, aiming to reduce hospital stays, improve recovery rates, and enhance overall patient care [18].

## Conclusions

Our case report highlights that open resection for recurrent lung abscesses in pediatric patients is an effective therapeutic approach when there is a failure with medical management alone. This technique, characterized by its simplicity and safety, offers significant advantages in terms of clinical outcomes and cost-effectiveness. Through our case report and literature review, we observed that patients undergoing open resection for recurrent lung abscess experienced marked improvements in both short- and medium-term health outcomes. The procedure was associated with a low rate of postoperative complications, facilitating rapid recovery and reducing the burden of prolonged medical management often required for recurrent infections. Our findings suggest that open resection should be considered a viable option in the management of recurrent lung abscesses, especially in pediatric populations where the recurrence of infection can significantly impact growth, development, and overall well-being.
